# Mathematical modeling of the dynamic storage of iron in ferritin

**DOI:** 10.1186/1752-0509-4-147

**Published:** 2010-11-03

**Authors:** J Cristian Salgado, Alvaro Olivera-Nappa, Ziomara P Gerdtzen, Victoria Tapia, Elizabeth C Theil, Carlos Conca, Marco T Nuñez

**Affiliations:** 1 Laboratory of Process Modeling and Distributed Computing, Department of Chemical Engineering and Biotechnology, University of Chile, Santiago, Chile; 2 Centre for Biochemical Engineering and Biotechnology, Department of Chemical Engineering and Biotechnology, University of Chile, Santiago, Chile; 3 Department of Biology, University of Chile, Santiago, Chile; 4 Council on BioIron at Children's Hospital Oakland Research Institute, 5700 Martin Luther King, Jr. Way, Oakland, CA 94609; 5 Department of Nutritional Sciences and Toxicology, University of California, Berkeley, CA 94720; 6 Department of Mathematical Engineering, Centre for Mathematical Modeling, University of Chile, Santiago, Chile; 7 Millennium Institute for Cell Dynamics and Biotechnology: a Centre for Systems Biology, University of Chile, Santiago, Chile

## Abstract

**Background:**

Iron is essential for the maintenance of basic cellular processes. In the regulation of its cellular levels, ferritin acts as the main intracellular iron storage protein. In this work we present a mathematical model for the dynamics of iron storage in ferritin during the process of intestinal iron absorption. A set of differential equations were established considering kinetic expressions for the main reactions and mass balances for ferritin, iron and a discrete population of ferritin species defined by their respective iron content.

**Results:**

Simulation results showing the evolution of ferritin iron content following a pulse of iron were compared with experimental data for ferritin iron distribution obtained with purified ferritin incubated *in vitro *with different iron levels. Distinctive features observed experimentally were successfully captured by the model, namely the distribution pattern of iron into ferritin protein nanocages with different iron content and the role of ferritin as a controller of the cytosolic labile iron pool (cLIP). Ferritin stabilizes the cLIP for a wide range of total intracellular iron concentrations, but the model predicts an exponential increment of the cLIP at an iron content > 2,500 Fe/ferritin protein cage, when the storage capacity of ferritin is exceeded.

**Conclusions:**

The results presented support the role of ferritin as an iron buffer in a cellular system. Moreover, the model predicts desirable characteristics for a buffer protein such as effective removal of excess iron, which keeps intracellular cLIP levels approximately constant even when large perturbations are introduced, and a freely available source of iron under iron starvation. In addition, the simulated dynamics of the iron removal process are extremely fast, with ferritin acting as a first defense against dangerous iron fluctuations and providing the time required by the cell to activate slower transcriptional regulation mechanisms and adapt to iron stress conditions. In summary, the model captures the complexity of the iron-ferritin equilibrium, and can be used for further theoretical exploration of the role of ferritin in the regulation of intracellular labile iron levels and, in particular, as a relevant regulator of transepithelial iron transport during the process of intestinal iron absorption.

## Background

The maintenance of body iron homeostasis is the result of a delicate balance between intestinal absorption and obligatory losses. Because of the lack of a specific excretory system, iron losses are mostly associated with bleeding and tissue sloughing, which, in healthy individuals, are compensated by similar amounts of iron absorbed in the duodenum. Traditionally, intestinal iron absorption is divided into three steps, named the uptake, intracellular and transfer phases. The uptake phase involves the transport of food iron from the intestinal lumen into the enterocyte cytoplasm by membrane transporters such as heme carrier protein 1 (HCP1, SLC46A1) [1] and divalent metal transporter 1 (DMT1, SLC11A2) [2,3], while the transfer phase involves the transport of intracellular iron from the cell cytoplasm into the blood circulation, a process mediated by the iron-export transporter ferroportin1 (SLC40A1) [4-6].

Little is known about the intracellular step, although 80-90% of the incoming iron is retained intracellularly [7,8]. Newly entering iron integrates into a cytosolic pool of weakly bound iron called the cytosolic labile iron pool (cLIP), constituted by iron bound to substances such as phosphate, nucleotides and amino groups [9,10]. From the cLIP, iron is either transported into the blood circulation by ferroportin1 or distributed to mitochondria, ferritin and other iron-requiring proteins. Recently, poly (rC)-binding protein 1 (PCBP1) was reported as a putative iron chaperone capable of delivering cytosolic iron to ferritin [11].

Ferritin is the only well characterized iron-storage protein in living organisms, widely distributed from archaea and bacteria to mammals [12]. The 24 subunits in maxi-ferritin form a hollow shell that can accept up to 4,500 *Fe*^3+ ^atoms in the form of crystallized diferric oxo-hydroxyl complexes [13]. Mini-ferritins (also called Dps proteins), found in bacteria and archaea, have 12 subunits but share many structural features with maxi-ferritins [12,14]. Ferritin plays a fundamental role in controlling cellular iron availability. It stores iron in an accessible and non-toxic form through a series of oxidation-reduction processes. The kinetics of iron entrance and exit have been characterized *in vitro*, with rate constants of 1,200 mol *Fe*/mol ferritin/second for the formation of the diferric peroxo complex [15] and 0.52 mol *Fe*/mol ferritin/second for exit from the protein [16]. Provided that the iron storage capacity is not exceeded, these data predict that iron released from ferritin should be quickly recaptured by the protein.

Ferritin is regulated by the iron regulatory protein/iron responsive element (IRP/IRE) mRNA system whereby high iron concentrations induce synthesis (reviewed in [17]). Ferritin synthesis is also transcriptionally regulated by pro-oxidants through an antioxidant response element (ARE) present in the 5'-untranslated region of L- and H-ferritin genes and a number of other genes such as thioredoxin reductase, NADPH quinine oxidoreductase and heme oxygenase; Bach 1 represses ARE genes and is derepressed by heme-Bach 1 interactions [18-20].

In intestinal cells, as in all cells, ferritin concentrations are regulated by iron dependent control of protein synthesis and degradation [21-23]. Data show that absorbed iron not exported from the enterocyte is stored in ferritin, but the recent awareness that, in solution at least, ferritin protein is a dynamic manager of iron, suggests the possibility of a dynamic role for ferritin during the process of intestinal iron absorption that has been little considered. Ferritin may play a role in the retention of absorbed iron, since under conditions of iron-deficiency, when ferritin iron concentrations and the total amount of ferritin protein cage is low, most of the absorbed iron passes directly from the cell to the plasma. In contrast, under iron repleted conditions a considerable part of the absorbed iron is retained in ferritin, some of which is lost into faecal excretion from epithelia sloughing [24,25].

A continuous source of iron to the cLIP derives from ferritin turnover. In Caco-2 intestinal cells, reported values for ferritin concentration and ferritin half-life are 1.1 pmol per mg of cell protein and 16 h, respectively [26]. Considering a mean load of 1,000 iron atoms per molecule of ferritin, turnover will provide about 68 pmol of iron per mg protein per hour, an amount comparable to the uptake of 30 pmol/mg protein/h by iron-starving Caco-2 cells [27]. Thus, during the process of intestinal iron absorption there can be an exchange between iron that enters the cLIP, iron utilized by the mitochondria and iron-containing proteins, iron that comes from ferritin turnover, iron that is captured by ferritin and iron that is transported to the blood plasma.

At present, there is no mathematical model that describes potential contributions of ferritin in controlling the cLIP during the process of intestinal absorption. Previously, a mathematical compartment model was generated to account for the three phases of intestinal iron absorption in patients with hereditary hemochromatosis. The model predicted that the transfer of iron from the cell into the blood was the main site of regulation in these patients [28], but ferritin was not considered to be a dynamic interactor and controller of the cLIP and ferritin mechanism was not fully taken into account.

In this work, we present a novel mathematical model to study the dynamics of iron storage and distribution in ferritin and to reveal its relevance in the process of cytosolic iron management in Caco-2 cells during intestinal iron absorption.

## Results and Discussion

### Standard ferritin sedimentation curve

**Figure 1:**
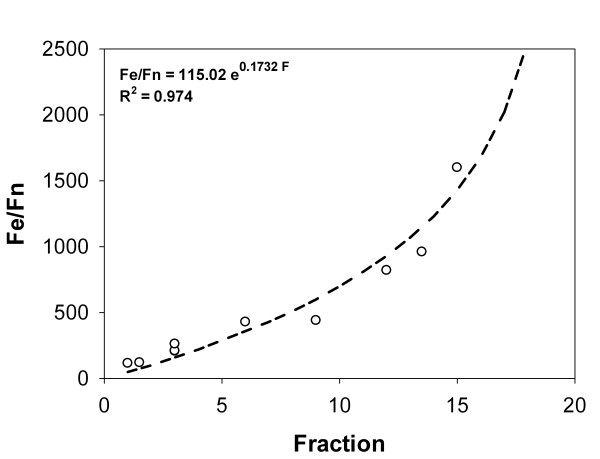
**Experimental standard calibration curve for the iron-to-ferritin ratio as a function of the fraction collected in a sucrose gradient**. Experimental standard calibration curve for the iron-to-ferritin ratio as a function of the fraction collected in a sucrose gradient. Horse spleen apoferritin was loaded with different amounts of iron [44], centrifuged and loaded into a sucrose gradient (1-25%). Fractions were collected and ferritin content was determined by the BCA assay. Circles correspond to experimental data points and the calibration curve is shown as a dashed line.

In the Supplementary Material (Additional file 1: Supplemental Figure S1), the sedimentation properties of ferritin with different iron contents in a 1-25% sucrose density gradient are shown. Clearly, ferritins with higher amounts of iron migrate towards a higher density zone of the gradient. However, the relationship between iron content and sedimentation follows a non-linear relationship. This sedimentation pattern should be the result of changes in particle density and particle diameter as reported [29]. The mode of the protein distribution for a given iron load was chosen as representative of its iron content, resulting in the calibration curve shown in Figure 1.

### Steady states

**Figure 2:**
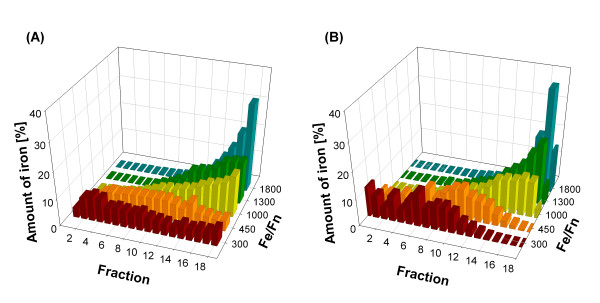
**Steady state distribution of iron content in ferritin**. **(A) ***In vitro *steady state distribution of iron content in ferritin. Horse spleen apoferritin was loaded with different iron to ferritin ratios of 300:1, 450:1, 1000:1, 1300:1 and 1800:1 [44] and loaded into a sucrose gradient (1-25%). Fractions were collected and ferritin content was determined by the BCA assay. The iron content was calculated using the calibration curve in Figure 1; **(B) **Simulation results for the steady state distribution of iron content in ferritin for iron to ferritin ratios of 300:1, 450:1, 1000:1, 1300:1 and 1800:1. The iron content distribution was transformed to sedimentation fractions using the calibration curve in Figure 1, allowing for the recovery of the distributions observed experimentally. Iron packages were transformed to iron atoms (50 atoms per package) and then associated to a corresponding experimental fraction.

The kinetic mathematical model was initially tested for its ability to recover the experimental steady states of iron load distribution in ferritin protein cages. Figure 2a shows the distribution of ferritin protein cages in the sucrose gradient after being loaded with different iron-to-protein contents, ranging from a small amount of iron (300:1 iron/ferritin) to a system closer to empirical saturation (1800:1 iron/ferritin). As the iron content in ferritin increased, the protein sedimented into more dense fractions. Interestingly, sedimentation developed into broad peaks, an indication that the *in vitro *iron loading conditions used produced an heterogeneous mineralization of the ferritin protein cages, as observed *in vivo *[30]. Results in Figure 2a show that the fraction of protein cages with a high iron content increases as the total amount of iron in the system increases, *i.e*. increasing the amount of iron in the system shifts the distribution of iron on ferritins to the right. Ferritin from Caco-2 cells cultured with varied amounts of iron in the culture media also yielded broad sucrose sedimentation patterns, with an average content of about 1,000 *Fe*/ferritin protein cage. Increasing iron concentrations in cultured cells were accommodated in part by increased ferritin subunit synthesis (Additional file 1: Supplemental Figure S1). These results suggest that an iron-to-ferritin ratio of 1:1000 is optimal for the iron buffering capacity of ferritin in cultured cells, which is close to the average iron content of many plant and animal ferritins.

The steady states achieved when the experimental conditions were used to simulate the model are shown in Figure 2b. Simulations were performed using parameters and initial conditions shown in Table 1. Ferritin levels were kept constant and the total amount of iron was set according to the experimental iron to ferritin ratios (300:1, 450:1, 1000:1, 1300:1 and 1800:1). The simulation results, after a non linear transformation given by the calibration curve, are very similar to the distributions observed experimentally. In particular the overall shape of the iron distribution is captured by the model.

**Table 1:** Mathematical model parameters

Parameter	Value	Unit	Description/comment
General			
*Fe_pack_*	50	atom packages^-1^	Number of atoms in an iron package (Arbitrary)
*Fn_max_*	4500	Atom	Maximum iron storage capacity in a ferritin protein cage [43]
$Fn_{max}^{eff}$	2500	Atom	Maximum measured iron storage capacity for a ferritin
*N*	50	packages	Maximum number of iron atoms packages that a ferritin protein cage can store *Fn_max_/Fe_pack_*
			
Kinetic constants			
*k_aso_*	1.6955 × 10^15^	pmol-packages^-1 ^hr^-1 ^cell^-1^	Association constant [15]
*k_dis_*	8.25192 × 10^7^	hr^-1^	Dissociation constant, calculated from *k_App _*[46]
*k_cat_*	7.776 × 10^6^	hr^-1^	Catalytic constant [46]
*k_d_*	4.33 × 10^-2^	hr^-1^	Proteolytic degradation constant [26]
${\tilde{k}}_{loss}$	2.56 × 10^7^	hr^-1^	Iron release constant: base value (see Figure 8 and text)
			
Initial conditions			
*Fn_total_*	5.52 × 10^-7^	pmol-ferritin cell^-1^	Total ferritin in a cell ∀*t *[15]
*Fe*	2.7880 × 10^-5^	pmol-packages cell^-1^	Initial intracellular iron concentration *t *= 0 [15]
*Fn_i_*	0	pmol-ferritin cell^-1^	∀*i *≠ 0
*Fn_0_*	*nFn_total_*	pmol-ferritin cell^-1^	All ferritin protein cages are empty at *t *= 0
$C_{Fn_{i}Fe}$	0	pmol-ferritin cell^-1^	There is no intermediary complex at *t *= 0

**Figure 3:**
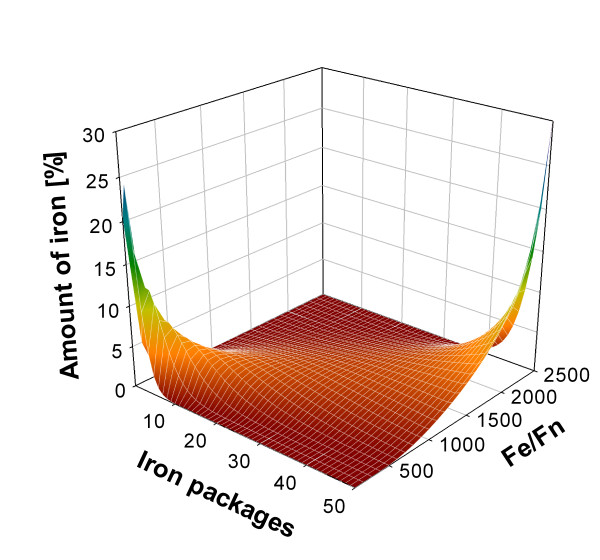
**Simulation results for the steady state distributions for a set of iron-to-ferritin ratios**. Simulation results for the steady state distributions for a set of iron-to-ferritin ratios ranging from 100:1 to 2500:1. A soft transition between steady state distributions from low to high iron to ferritin ratios is observed.

Figure 3 shows simulated steady state distributions for a number of iron to ferritin ratios ranging from 100:1 to 2500:1. Simulation results show that as the iron-to-ferritin ratio is increased, there is a soft transition between steady state distributions from low to high iron-to-ferritin ratios. This result is in agreement with experimental data indicating that the model is able to capture steady state iron content distribution in ferritin *in vitro*. This suggests that regulation of the iron-ferritin system could be modelled as a control system designed to keep a given iron-to-ferritin ratio. This would allow the system to maintain a controlled cytosolic cLIP. For example, in DU145 cells, the cLIP was decreased in cells pretreated with the iron chelator desferrioxamine or when ferritin synthesis was inhibited by TNF treatment, which induced a drop in the amount of ferritin light chains [31]. Such a ferritin feedback loop where iron and oxygen are both regulatory signals for ferritin synthesis and substrates consumed in ferritin protein mineralization has recently been described [23].

### Dynamic behavior

**Figure 4:**
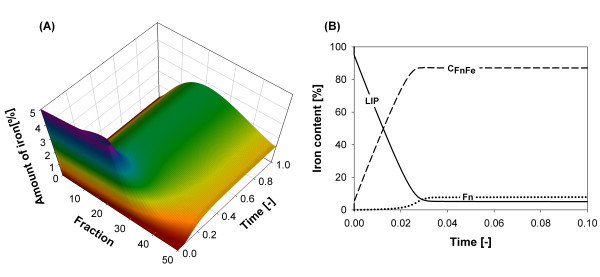
**Simulation results for the dynamic evolution for ferritin's iron content**. Simulation results for the dynamic evolution of ferritin's iron content. An iron to ferritin ratio of 1000:1 was considered. **(A) **Distribution of iron stored in ferritin over time. A fast transient is observed at the beginning of the simulation, along with the broadening of the iron distribution in ferritin over time; **(B) **Iron content profiles over time for cLIP and ferritin species for early simulation times.

The dynamic behavior of the model was studied for an iron to ferritin ratio of 1000:1 to be consistent with experimental data [32], (Additional file 1: Supplemental Figure S1). Figure 4 shows the iron content distribution in ferritin over time. Figure 4a shows that after an iron influx pulse, iron is initially more abundant in the cLIP and it is quickly taken-up to produce an abundant initial pool of low iron content ferritins, because empty ferritin protein cages use their multiple active sites (pores) to capture ferrous irons (2/pore), creating an initial mineral core. From then on, ferritins follow the mechanism previously proposed, *i.e*. they would either loose or capture (mineralize) a package of iron, or degrade, releasing all their iron content [33]. All these mechanisms working together simultaneously lead to an increase in the heterogeneity of ferritin's iron content in time, causing a shift in its distribution. In fact, as time progresses, the average ferritin iron content increases, as well as the number of ferritins operating at maximum capacity, conditions that occur *in vivo *during iron overload and which are accompanied by the formation of a degraded, toxic product called hemosiderin. The shift of the iron distribution over time shown in Figure 4 illustrates this enrichment process.

The amount of time required by the simulated model to reach steady state was determined to be in the order of 1 × 10^-4 ^h. Diffusional phenomena are not considered since a perfectly mixed system was assumed. For the sake of simplicity and comparability, all simulation results were normalized to the time when the respective system reaches steady state.

Simulation results shown in Figures 4a and 4b indicate that three distinct stages can be identified in ferritin iron mineralization. Figure 4a illustrates how the distribution of iron stored in ferritin changes over time. Figure 4b shows the total iron in the cLIP and ferritin species as a function of time for early simulation times.

**Stage 1**: The first stage corresponds to iron uptake by ferritin which extends until 0.03 units of nondimensional simulation time. As shown in Figure 4b this process exhibits a fast dynamic, causing as a main consequence that the steady state for the cLIP is achieved early in the simulation. During this period, the iron distribution and average iron content in ferritin also changes rapidly (Figure 4a). In addition, the fraction of total cell iron in ferritin (*C_FnFe _*+ *Fn*) increases, reflecting the relative magnitude of the kinetic constants in the association-dissociation equilibrium, *k_aso_*/*k_dis _*≈ 2 × 10^7^.

**Stage 2**: From 0.03 to 0.4 units of simulation time, the total amount of iron captured by ferritin and the cLIP stabilizes (Figure 4b). However, as shown in Figure 4a, the distribution of iron stored in ferritin continues to change, although it does so more slowly than in the first stage. The distribution shape broadens in this stage, accounting for an increase in the diversity of iron content in the population of ferritin protein cages.

**Stage 3**: At 0.4 units of the nondimensional simulation time, a final stage develops. In this phase, the iron distribution in ferritin changes much more slowly, reaching the final steady state at 1.0 units of nondimentional simulation time (shown in Figure 4a).

### LIP control

**Figure 5:**
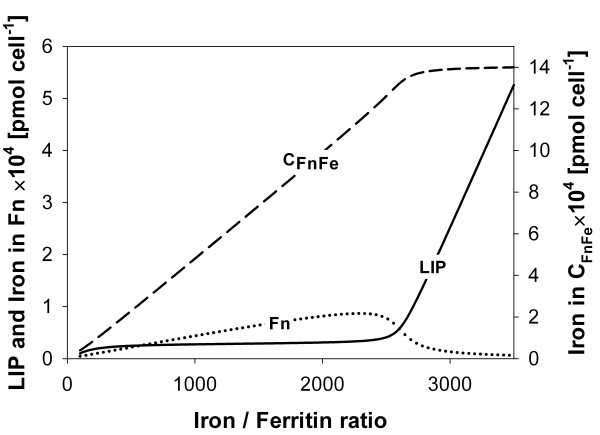
**Simulation results for the iron content in ferritin and in the cLIP**. Simulation results for the iron content in ferritin species and in the cLIP at the steady state reached by the model system starting from different iron to ferritin ratios.

It has been proposed that ferritin plays the role of an iron buffer in the cell [13]. The simulations described here support and supplement such a model. Figure 5 shows the iron amount in the cLIP and ferritin species at the steady state achieved by the model system in simulations starting from different iron-to-ferritin ratios. Notice that when the iron-to-ferritin cage ratio is greater than the maximum iron capacity of ferritin ($Fn_{max}^{eff}$), iron accumulates in the cLIP. *In vivo*, hemosiderin, which is an insoluble toxic degradation product of ferritin from which iron can be leached out into the cLIP, begins to appear at this stage.

The model also predicts that when the iron-to-ferritin ratio is in the operational range (100:1-2500:1) the cLIP remains almost constant. In fact, in the range from 500:1 to 2000:1, the slope of the cLIP in Figure 5 is such that a change of 100 units in the iron to ferritin ratio causes an average change of only 1.3% with respect to the reference value. This indicates that ferritin acts indeed as a buffer, keeping the amount of iron in the cLIP essentially independent from the iron-to-ferritin ratio. Such a buffering capacity is crucial in cellular systems and emphasizes the relevant contribution of the genetic endproducts of the cellular iron homeostatic system, ferritin protein cages, which act through consumption and stabilization of the regulatory signal (iron) during mineralization. In the face of rapid changes in cellular iron content ferritin activity provides the first quick cellular iron homeostatic response, which can be subsequently followed by the activation of slower regulatory processes at the transcriptional or translational levels which have a response time-scale several orders of magnitude larger than enzyme kinetics.

An interesting observation can be extracted from Figure 5 for iron-to-ferritin ratios under 500:1. In fact, it is evident that the amount of iron in the cLIP shifts down for these low iron (iron starvation) conditions. However, the ferroxidase activity of ferritin is not turned off during iron starvation conditions. Ferritin retains the same catalytic activity in all simulation experiments along the entire range of iron concentrations. The observed behavior is due to the continuous and dynamic nature of the iron capture and release processes that transport iron into and from the ferritin mineral core, which do not have a fixed steady-state equilibrium point with respect to the total iron amount. As a result, the dynamics of the ferritin system leaves the amount of iron in the cLIP to vary directly as a function of the total amount of iron available in the system at low iron concentrations (iron starvation). Interestingly, the ferritin iron storage system lets the cytoplasmic iron pool grow as the amount of iron available for the cell increases, but always keeping it below a certain threshold concentration. When the iron concentration becomes excessive and possibly dangerous, the ferritin dynamic storage system is able to tightly control the amount of iron in the cLIP and prevent iron from reaching toxic concentrations by keeping the cLIP at an essentially constant level (around 3 × 10^-5 ^pmol/cell), but iron is left almost freely available for the cell at lower concentrations. Hence, no oxidative damage will occur inside the cell, because the amount of iron in the cLIP is always kept at very low levels, both at low and high iron concentrations.

Experimental results show that the cLIP increases transiently following an iron challenge, but the change is homeostatic in nature. However, this homeostatic capacity is surpassed in the case of iron overload, which results in an increased cLIP that is able to initiate oxidative damage cascades [9]. The model predicts that ferritin progressively looses its iron buffering capacity when the iron to ferritin ratio increases beyond 2,500. This occurs because these conditions imply a saturation of ferritin protein cages with iron. Hence, if more iron enters the system, ferritin is unable to capture the excess iron atoms and the cLIP increases. This is a saturation behavior that is typical for all chemical and biological buffers and is in agreement with experimental observations.

**Figure 6:**
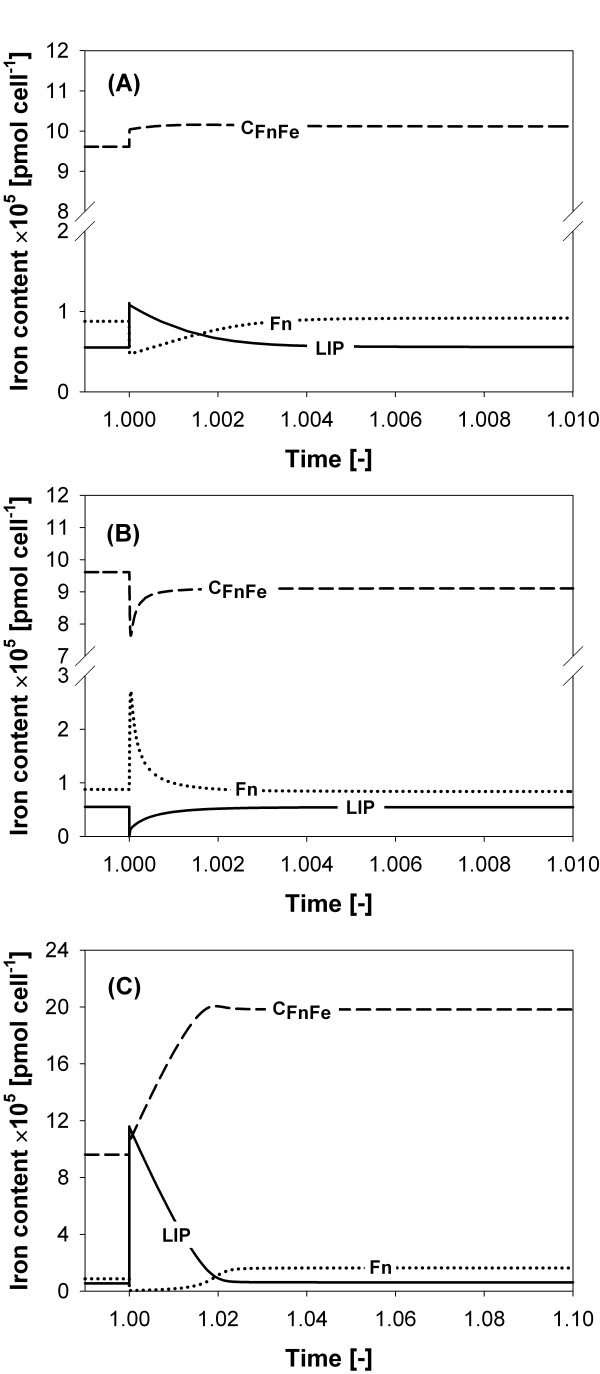
**Simulation results for the iron content profiles over time**. Simulation results for the iron content profiles over time for cLIP and ferritin species: **(A) **when a pulse of free iron is introduced into the system at *t *= 1.0 simulation time units. The pulse of iron given to the system was equal to the steady state amount of iron in the cLIP; **(B) **when all iron in the cLIP is washed out of the system at *t *= 1.0 simulation time units; **(C) **when a pulse of free iron is introduced into the system at *t *= 1.0 simulation time units. In this case the pulse of iron given to the system was equal to 20 times the steady state amount of iron in the cLIP, doubling the total iron in the system.

The dynamic behaviour of iron in the cLIP related to ferritin activity in response to a perturbation in the amount of labile iron is shown in Figure 6. These graphs show that the steady state amount of cLIP is essentially insensitive to the addition or removal of iron, due to the buffering capacity of ferritin. For example, doubling the amount of labile iron gives an increment of only 0.6% on the final steady state amount of cLIP (Figure 6a). Resistance to a simulated decrease (depletion) in the labile iron pool is also clear (see Figure 6b). Here, ferritin reacts by immediately releasing iron to the cLIP to reach a steady state which is only 0.7% less than the previous one. The effect on the cLIP and ferritin iron content after a violent unphysiological 20-fold increase in the cLIP is shown in Figure 6c. Here, the pulse of iron equals the initial iron given at *t *= 0, doubling the total iron in the system. After the perturbation, ferritin is able to immediately mineralize most of the extra iron. As a result, the amount of iron as cLIP in the new steady state is only 13.1% higher than the previous one.

### Qualitative and quantitative analysis of the kinetic constants

Whether simulating the steady state amount of cLIP or sudden changes in the cLIP, the simulation results show the high capacity and effectiveness of ferritin protein nanocages to quickly react and control changes in the amount of cLIP. The simulation results indicate that a given set of kinetic constants, specifically a given ratio between the kinetic constants, determines the setpoint control level of the cLIP, *i.e*. the amount of iron allowed to be present in the cLIP under normal cellular circumstances. The absolute value of those kinetic constants in the ferritin system is very high, which determines the fast buffer reaction of the system. In fact, the effectivity of the buffering system is exclusively based on the combination of fast and continuous iron uptake and release processes. If one of these processes is lost or its rate is too slow, the buffer function collapses, intracellular iron control becomes defective and the cell's probability of survival is diminished.

In this same way, it is interesting to analyze the values of the kinetic constants to gain insights about other proposed ferritin functions. In particular, ferritin has always been associated with an iron storage function inside the cell. Indeed, the model simulations show that ferritin is able to perform such a function, but this role does not require values of the iron entrance constants, *i.e*. *k_aso_*, *k_dis _*and *k_cat _*, as high as those found in ferritin. In fact, much smaller rates should be enough to ensure a correct iron storage function.

Ferritin has also been associated with an antioxidant function, which is also supported by the fact that its synthesis is activated through regulators associated with oxidative stress [18-20]. Ferritin prevents oxidative damage by sequestering pro-oxidant and free-radical forming iron ions and dioxygen (or in the case of Dps, mini-ferritins, hydrogen peroxide) from the cytoplasm and keeping them in an inactive form. When analyzing the kinetic rates associated with ferritin, it is interesting to note that high iron release rates from the inactive mineral core of ferritin protein cages are probably unfavorable for this antioxidant activity, unless the pores are held closed by a molecular clamping mechanism (regulatory protein) when iron is in excess. Hence, a protein with a lower or no iron release rate should be better than ferritin itself to control oxidative processes associated with high iron concentrations.

Therefore, the particular combination of measured high kinetic constant rates of ferritin, both for iron uptake and release, seems to have evolved primarily to create a powerful intracellular iron buffering system. In comparison, the iron storage and antioxidant functions of ferritin are not optimally evolved, but seem to be subordinated side-effects of the buffer function. Thus, contemporary ferritins probably evolved as a multifunction protein to perform a major iron buffering inside the eukaryotic cell, retaining secondary iron storage and antioxidant roles. In some bacteria separate genes encode ferritins that respond either to oxidant or iron stresses; the role of ferritin as an antioxidant appears to be especially important in human pathogenic bacteria and in anaerobic archea.

## Conclusions

A mathematical model based on the structure/function of ferritin protein nanocages and purely kinetic considerations about the mineralization/dissolution of the ferritin iron mineral core was proposed to elucidate functional characteristics of the dynamic behavior of ferritin *in vitro *and *in vivo*. Kinetic parameters and enzymatic constants for the model were directly taken or calculated from published works. Simulation results were compared to steady state distributions of iron in ferritin obtained experimentally. The experimental results effectively showed a non-uniform distribution of iron content in the population of ferritin protein cages, *i.e*. all ferritin cages do not contain the same amount of iron, even though the initial ferritin population is homogeneous (unloaded apoferritin). The model was able to capture the main features of the experimental distributions, validating the feasibility of the mechanistic structure proposed and parameters used.

Specifically, the model predicts that the main function of ferritin is to operate as an extremely fast, high-capacity, iron buffer that maintains mobile iron concentrations (cLIP) until transcriptional or translational regulatory processes occur (*e.g*. changes in ferritin synthesis). In this sense, the model indicates that the mineralization/demineralization process effected by ferritin is able to maintain intracellular cLIP levels approximately constant, even when large perturbations in intracellular iron levels are introduced.

The ferritin iron buffering system operates effectively in a broad iron-to-ferritin range with high robustness. Interestingly, ferritin does not sequester iron in iron starvation conditions, but tightly controls iron availability when iron is in excess, which demonstrates the fine-tuning and effectivity of the ferritin iron buffer system. The analysis of the magnitude of the kinetic parameters of the ferritin kinetic model indicated that iron buffering and cLIP stabilization are possibly the main functions of ferritin in the cell, and iron storage and antioxidant functions are arguably subordinate functions derived from the main buffering activity.

Finally, model simulations showed that the iron uptake process by ferritin is extremely fast, which is in agreement with experimental observations, but iron release was shown to be equally fast when pore folding provides no impedance as observed in recombinant ferritins in solution [14,34]. These fast equilibrium mechanisms are able to provide immediate protection from abrupt changes in iron concentrations during the period when slower responses to stress such as transcription and/or translation are activated.

## Methods

### The kinetic model

Ferritin quaternary structure comprises 24 α-helix protein subunits forming a characteristic nanocage that encloses an inner cavity completely isolated from the external medium [13]. Inside this isolated cavity, ferritin stores iron as insoluble *Fe*^3+ ^species (ferrihydrite crystallites) by sequestering *Fe*^2+ ^from the external medium. When entering the ferritin protein nanocage, ferrous ions pass through any of eight pores formed by three subunits at each three-fold symmetry axis of the protein shell. After this entrance, *Fe*^2+ ^is oxidized to *Fe*^3+ ^at diiron catalytic sites followed by electron transfer to dioxygen (premineral, diferric oxo) translocation and mineral nucleation inside the internal cavity [13,35,36].

**Figure 7:**
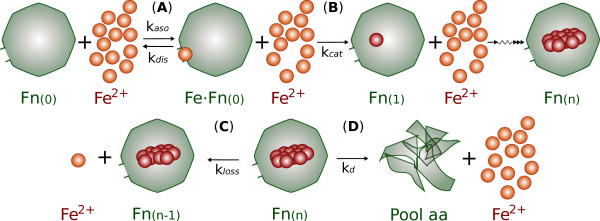
**Proposed mechanism for iron uptake/release by ferritin**. Proposed mechanism for iron uptake/release by ferritin. **(A) **Iron (*Fe*) enters through a pore in the apoferritin protein cage structure (*Fn*_(0)_), forming an iron-ferritin complex (*FeFn_0_*) through a reversible process. **(B) **A series of redox reactions lead to iron incorporation into the mineral core of ferritin (*Fn*_(1)_). After n incorporation steps, the Ferritin species *Fn*_(*n*) _is obtained **(C) **Iron can be released from the mineral core through redox and transport processes. **(D) **Ferritin molecules can also be proteolytically degraded, returning their entire iron content to the cLIP during this process.

Ferritin iron mineralization as a catalytic mechanism (see Figure 7), namely as a ferroxidase (EC 1.16.3.1), can be described applying usual enzymology mathematical tools, even though the process by which ferritins synthesize mineral and release iron is highly complex and not entirely understood [34].

In recent years, stopped-flow experiments with a transient peroxodiFe(III) formed at the ferroxidase active site of ferritin have revealed very unusual kinetic curves, dependent on the iron-to-protein ratio [37]. A mathematical model for the kinetics of the catalytic process has been previously proposed [38]. The model correctly explains the unusual kinetic behavior of the enzyme and the time course of the appearance/disappearance of the reaction intermediates. This kinetic model for catalysis includes two sequential mechanisms, dependent on the amount of iron available to the protein and the concentration of electron acceptors (dioxygen) for the oxidation step, and a new intermediate in the catalytic reaction (a putative hydroperoxodiFe(III) complex). However complex the catalytic mechanism is, the formation of the mineral core, *i.e*. the final product of the enzymatic reaction, can be described more easily considering the ferritin mineralization mechanism as a serial process for a single chemical species (iron) characterized by an initial affinity equilibrium step and subsequent consecutive monomolecular reactions. These conditions imply that the global enzymatic reaction can be described by a Michaelis-Menten-like mechanism with association, dissociation and catalytic processes characterized by an apparent kinetic constant, respectively *k_aso_*, *k_dis _*and *k_cat _*(see Figure 7). In this mechanism, *k_aso_*/*k_dis _*represent the affinity equilibrium constant of the pores of one ferritin protein cage for external *Fe*^2+ ^and *k_cat _*encompasses the oxidation, translocation and precipitation rates in the pores. In fact, *k_cat _*takes the value of the rate constant of the global process rate limiting step. Electron transfer steps can be considered fast processes compared to ion translocation and precipitation, given the relative abundance of cytoplasmic electron acceptors (oxygen) in normal conditions. Therefore, *k_cat _*can be considered at least initially as independent from the oxidation step and so the concentration of electron acceptors was not included in the kinetic model.

Though the ferritin kinetic mechanism includes a Michaelis-Menten enzymatic reaction, there is a fundamental difference from most models: the reaction product (diferric oxo) is not released back to the external medium, but it is transferred into the internal cavity of the catalytic protein cage. This implies that the enzyme, *i.e*. ferritin, is modified by the reaction by increasing its iron content. In this way, a population of different enzymes (ferritin protein cages) with different iron contents is created, but each nanocage retains its catalytic activity intact, because the pores remain unblocked (maintaining the same *k_aso_*, *k_dis _*and *k_cat _*independently from the size of the iron mineral core) unless the protein cage becomes full of iron atoms. In this latter case, there is no iron translocation and mineralization, *i.e*. *k_cat _*= 0. Therefore, the iron entrance mechanism can be described by the following kinetic equations defined for each different ferritin species *Fn_i _*containing *i *iron atoms inside its protein nanocage:

(1) $$Fn_{i}+Fe\overset{k_{aso}/k_{dis}}{\leftarrow }C_{Fn_{i}Fe}\overset{k_{cat}}{\to }\;Fn_{i}{}_{+1}\;\forall i=0,..,N-1$$

(2) $$Fn_{i}+Fe\overset{k_{aso}/k_{dis}}{\leftarrow }C_{Fn_{i}Fe}\;\forall i=N$$

Ferritin can also release iron through nanocage pores, but this process is less understood than iron uptake. It is clear that rates of iron loss will depend on the accessible surface of the mineral core. The process also depends on the presence of reductants and on complex structural changes of the pores between folded and unfolded states that can affect iron release rates [12,14]. As the mechanism of iron release is not completely understood, a first order kinetic equation was proposed to model the loss of one iron atom from the mineral core as:

(3) $$Fn_{i}\overset{k_{l}{}_{oss,i}}{\to }Fn_{i-1}+Fe\;\forall i\neq 0$$

Even though *k_loss,i _*is initially defined as a constant, it can be dependent on other variables such as the iron content of the mineral core and the pore availability and folding state. These functional dependencies of *k_loss,i _*will be explored and determined below based on the comparative analysis of model simulations and experimental results.

*In vivo*, ferritin can also be proteolytically degraded by lysosomal or proteasomal degradation. When the ferritin protein cage is degraded to its amino acid components, the iron of the mineral core is reduced and returned to the cLIP [33,39] and amino acids are recycled back to the cytoplasm. In Caco-2 intestinal cells, ferritin has a half-life of 16 h [26]. Therefore, a first order kinetic equation, characterized by a kinetic constant, *k_d_*, can be proposed for this proteolytic degradation process regardless of its specific mechanism:

(4) $$Fn_{i}\overset{k_{d}}{\to }aa_{Pool}+i\;Fe\;\forall i=0,..,N$$

where *aa_Pool _*represents the cytoplasmic amino acid pool. *In vivo*, ferritin subunits can also be synthesized *de novo *from the cytoplasmic amino acid pool. This complex process can also be represented by a first order synthesis rate:

(5) $$aa_{Pool}\overset{k_{synt}}{\to }Fn_{0}$$

In this work, we are interested in the simulation of the fast stages of the intracellular iron regulation process mediated by ferritin in short periods of time. In these short time spans, the total amount of ferritin can be assumed to be constant, because the time scale of regulation is much slower. This is a standard quasi-steady-state assumption which comes from the way we have separated time scales for synthesis and degradation; in doing so, we have followed usual biochemistry rules as explained in [40]. Hence, to preserve the total amount of ferritin, the amount of degraded and synthesized ferritin must be equal. That is

(6) $$v_{synt}=v_{deg}$$

Therefore, equations (4) and (5) become

(7) $$Fn_{i}\overset{k_{d}}{\to }Fn_{0}+i\;Fe\;\forall i$$

Notice that according to Eq. (7) there is no change in the amount of *Fn*_0 _due to degradation since degradation and synthesis processes occur at the same rate. Given the order of magnitude of *k_d _*with respect to the other parameters in the model, the effect of removing Eq. (7) is not significant in this scenario, where regulation of ferritin expression levels is not considered. In fact, eliminating this flux resulted in a change of less than 1.5 × 10^-3^% in all the system's variables (data not shown). However, this terms were kept in the model in order to provide a more general framework, suitable for the inclusion of regulatory factors later on.

Finally, the kinetic model is represented by the following equations:

(8) $$Fn_{i}+Fe\overset{k_{aso}}{\to }C_{Fn_{i}Fe}\;\forall i=0,..,N$$

(9) $$C_{Fn_{i}Fe}\overset{k_{dis}}{\to }Fn_{i}+Fe\;\forall i=0,..,N$$

(10) $$C_{Fn_{i}Fe}\overset{k_{cat}}{\to }Fn_{i+1}\;\;\forall i\neq N$$

(11) $$Fn_{i}\overset{k_{loss,i}}{\to }Fn_{i-1}+Fe\;\forall i\neq 0$$

(12) $$Fn_{i}\overset{k_{d}}{\to }Fn_{0}+i\;Fe\;\forall i=0,..,N$$

### The mathematical model

The kinetic system presented in the previous section has three state variables: *Fn_i_*, $C_{Fn_{i}Fe}$ and *Fe *with *i *= 0, .., *N*. In order to study the dynamics of the kinetic model described by equations (8) to (12) a dynamic model was developed. This model was built performing a mass balance over the system's species and using mass action kinetic relationships. The final model consists of the following system of ordinary differential equations:

Iron mass balance

(13) dFedt=+kdis∑i=0NCFniFe+kd∑i=0Ni⋅Fni +∑i=1Nkloss,i⋅Fni−kaso⋅Fe∑i=0NFni

Ferritin mass balance

(14) dFn0dt=+kdis⋅CFn0Fe+kd∑i=1NFni +kloss,1⋅Fn1−kaso⋅Fe⋅Fn0

(15) dFnidt=+kdis⋅CFniFe+kcat⋅CFni−1Fe +kloss,i+1⋅Fni+1−(kd+kaso⋅Fe)·Fni −kloss,i⋅Fni ∀i=1,..,N−1

(16) dFnNdt=+kdis⋅CFnNFe+kcat⋅CFnN−1Fe −(kd+kaso⋅Fe)⋅FnN−kloss,N⋅FnN

Ferritin-Iron complex mass balance

(17) dCFniFedt=+kaso⋅Fni⋅Fe −(kdis+kcat)⋅CFniFe ∀i=0,..,N−1

(18) $$\frac{dC_{Fn_{N}Fe}}{dt}=+k_{aso}\cdot Fn_{N}\cdot Fe-k_{dis}\cdot C_{Fn_{N}Fe}$$

This mathematical model has been conceived to represent the uptake or release of one iron atom at a time, which results in a system of 2*N *+ 3 differential equations. Mathematically, the same equation system can be equivalently used to represent the uptake of one iron atom package, *i.e*. a fixed number of iron atoms, at a time. The only difference is the loss in resolution caused by lumping iron in packages instead of treating atoms one by one. A suitable iron package size of 50 iron atoms per package was chosen to solve the differential equation system. Hence, the value of *N *used in the simulations of these work was decreased from 2,500 to 50, and the size of the equation system was lowered from 5,003 to 103 simultaneous equations with no significant loss of resolution with respect to the amount of iron contained in the mineral core.

**Figure 8:**
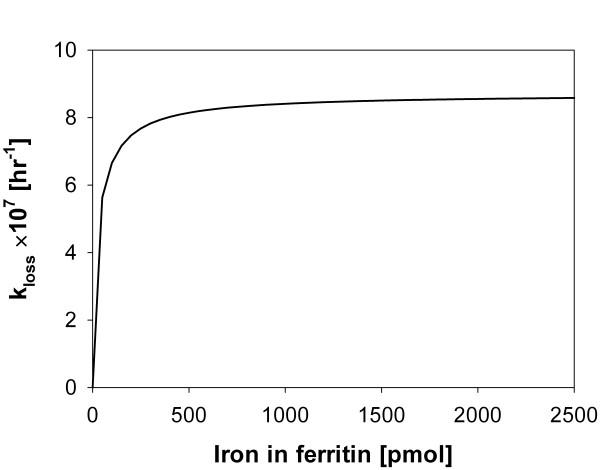
**Iron release kinetic constant (*k_loss_*) as a function of the number of iron atoms in ferritin**. This model considers a mechanism where the loss of iron is controlled by the iron content in ferritin at low iron levels and by the availability of exit pores at high iron levels.

Since this model does not consider diffusional or spatial distribution effects, this is a lumped component model. General mathematical results for this type of ordinary differential equations show that their steady states strongly and directly depend on the kinetic constant values and relative ratios (see, *e.g*., [41]), particularly on the values of the constants *k_loss,i _*with *i *= 0, .., *N *. Thus, the performance of the system to realistically model the global process depends on an appropriate choice of this set of constants. Certainly, a fundamental part of the construction of our model consisted in proposing a suitable functional dependency of *k_loss,i _*in terms of *i*. Table 1 shows a list of available values in the literature for all those constants that we have assumed to be independent from *i*. For the dependence of *k_loss,i _*in terms of the number *i *of iron packages stored in ferritin, we proposed an expression based on Hill's equation:

(19) $$k_{loss,i}=\{\begin{array}{ll} {\tilde{k}}_{loss} & \text{if }i=0,\\ {\tilde{k}}_{loss}\left(1+\frac{\kappa \cdot i^{n}}{\theta^{n}+i^{n}}\right) & \text{if }i\neq 0. \end{array}$$

where *n *= 1, θ = 1 and κ = 2.4 were chosen in order to recover the behavior of experimental results. A plot of *k_loss,i _*as a function of the number of iron atoms in ferritin (*Fe_pack_*) is shown in Figure 8. This expression was developed through a mathematical rationalization of biological intuitions about the system. In addition, similar equations have been shown to be good phenomenological approximations for biological rates [42].

It is clear that iron release rates should increase as the surface of the mineral increases and hence the iron release rate will reflect the size of the mineral core in each ferritin protein cage. Iron release from the ferritin mineral requires electron transfer to reduce the ferric to ferrous, hydration of the *Fe-O-Fe *bonds and migration of ferrous iron released from the mineral through the ferritin protein cage to the outside of the ferritin molecule, presumably to be trapped by iron carriers. While much of the molecular details of the process remain unknown it is clear that the folding/unfolding of the pores at the three-fold axis of ferritin protein cages influences the rate of iron removal [12,14]. Thus iron release rates are a combination of mineral dissolution rates, which reflect the amount of exposed surface of the mineral core, and of the folding state of the pores in the protein cage. Removal of iron in small minerals, estimated at 500 iron atoms for this model, has a different relationship to mineral size than for larger minerals, possibly because with larger minerals, there has been more dehydration in the iron core. Ferritin pore unfolding appears to influence transfer of electrons from the reductants, such as FNH_2 _or DTT. However, nothing is known about the relationship of the protein pore structure and reprotonation of the *Fe-O-Fe *bonds to release iron from the mineral.

A linear relationship with the form $k_{loss,i}={\tilde{k}}_{loss}\cdot i$ similar to a first order kinetic with respect to stored iron, proved to be too strong to represent the global iron release phenomenon (in the model simulations, all ferritins loose a fraction of the iron very rapidly). This mathematical expression does not consider the geometric relationship between the mass (volume) of the iron mineral core inside the protein cage and its exposed surface. In fact, for a given number of atoms *i *in the solid core inside a pseudo-spherical cage, it is easy to demonstrate mathematically that the exposed surface available for hydration and dissolution increases roughly as a function of *i*^2/3^, the value of which is always less than *i*.

The linear relationship does not take into account the fact that iron release from the central core occurs via the three-fold axis pores of the protein cage. Each 24-mer protein cage has eight pores that serve as gates for the release of iron from the mineral core. As the number of available exit channels ("active sites") for a single massive mineral core is fixed, simple kinetic considerations allow prediction of a saturation behavior expected to occur as a function of the amount of available iron atoms able to be released from the mineral core, which limits the global iron release rate from the protein cage. The maximal saturation rate could vary depending on additional factors, such as the folding/unfolding state of the pores, concentration of iron chelators and/or electron transfer to reduce ferric to ferrous ions.

Taken together, the iron release rate depends on (a) the area of exposed mineral core surface, (b) the degree of hydration of the mineral core, (c) the saturation of three-fold axis pores of the ferritin protein cage, (d) the concentration of chelators and (e) the concentration of reducers, in a mechanism wherein each factor affects a different kinetic process in a mostly sequential global mechanism wherein the rate-determining factor will be the slowest one. Taking into account these iron release mechanistic factors, the expression in Eq. (19) was proposed. This function captures the dependence of the iron release rate on the hydrated surface area of the mineral core when *i *is small (small mineral core) and a pore saturation behavior that becomes relevant for larger values of *i *(large mineral core). The maximal saturation rate is a constant that in turn will depend implicitly on the concentration of chelators and reducers and the gating behavior of the pores. This type of kinetic dependence of *k_loss _*as a function of the iron amount in the mineral core (*i*) allowed obtention of simulation results in agreement with the observed experimental results.

### Simulations

Simulations of the model (equations (13) to (18)) were performed in order to investigate the dynamic characteristics of the system. Parameters and initial conditions used in numerical simulations are summarized in Table 1. Briefly, the ODE system was simulated until steady state conditions were satisfied. Those conditions included a maximum number of iterations and a unnoticeable change in the system's variables along two consecutive time spans. As the ODE system was found to present stiff behaviour, a commercially available stiff ODE package was used to perform all simulations.

As shown in Table 1, all simulations were carried out using an initial condition with no *C_FnFe _*complex and empty apoferritin protein cages. The iron available for ferritin mineralization was provided by iron from the surrounding medium, *i.e*. the cLIP. Notice that even when the reported theorical iron capacity of ferritin is *Fn_max _*= 4500 iron atoms per ferritin [43], numerical simulations were performed using a lower value of $Fn_{max}^{eff}$ = 2500 iron atoms per ferritin, since our experimental results indicated that ferritin precipitates *in vitro *for a ratio *Fe/Fn *> 2500 (data not shown). Finally, the value of ${\tilde{k}}_{loss}$ was adjusted in order to obtain a cLIP corresponding to 5% of the total cell iron at steady state, which is coherent with previously reported values [9].

### Experimental setting: Velocity of sedimentation of ferritins with different iron contents in a sucrose gradient

Commercial horse spleen ferritin (Sigma Chem. Co.) was depleted of iron by treatment with thioglycolic acid [44] and loaded with different amounts of iron as described [45]. After iron loading, ferritins were resolved by gel filtration in a Sephacryl S400 column equilibrated in Hepes 0.15 M, NaCl 0.1 M, pH 7.0. The main peak was collected, its protein content was determined by the bicinchoninic acid (BCA) assay (Pierce, Rockford, Ill) and its iron content by absorbance at 420 nm [44]. To determine their migration properties, ferritins were loaded into a 5 ml 1-25% sucrose density gradient and centrifuged for 2.5 h at 112,000 × g in a Sorvall Combi ultracentrifuge equipped with an AH-850 rotor [45]. The gradients were fractionated in 250 *μ*L aliquots and ferritin content was determined by the BCA assay. A gradient sedimentation curve was constructed by plotting the position of each ferritin fraction in the gradient versus its iron content. This migration standard curve was later used to determine ferritin iron content in cell extracts and to model the iron uptake by ferritin *in vitro *and *in vivo*.

## List of abbreviations

*Fn_i _*[pmol-ferritin cell^-1^]: Mass of ferritin protein cages with *i *iron packages; *Fe *[pmol-packages cell^-1^]: Mass of iron packages; *C_FniFe _*[pmol-ferritin cell^-1^]: Mass of the intermediary complex of ferritin with *i *iron packages and iron; *aa_Pool _*[-]: Cytoplasmic amino acid pool; *i *[-]: Number of iron packages; *Fe_pack _*[atom packages ^-1^]: Number of atoms in a iron package (Arbitrary); *Fn_max _*[atom]: Maximum iron storage capacity for a ferritin; $Fn_{max}^{eff}$ [atom]: Maximum measured iron storage capacity in a ferritin protein cage; *N *[packages]: Maximum number of Fe atoms packages that a ferritin protein cage can store (*Fn_max_/Fe_pack_*); *k_aso _*[pmol-packages^-1 ^hr^-1 ^cell^-1^]: Association constant; *k_dis _*[hr^-1^]: Dissociation constant; *k_cat _*[hr^-1^]: Catalytic constant; *k_d _*[hr^-1^]: Proteolytic degradation constant; *k_loss,i _*[hr^-1^]: Iron release constant for a ferritin protein cage with *i *iron packages; ${\tilde{k}}_{loss}$ [hr^-1^]: Iron release constant: base value.

## Authors' contributions

JCS developed the mathematical model, programmed the algorithm, designed *in silico *experiments, performed biological interpretation of the results and drafted and revised the manuscript. AO-N proposed the core of the mathematical model and performed biological interpretation of the results. ZPG developed the mathematical model, designed *in silico *experiments and performed biological interpretation of simulations and experimental results. VC designed, setup and carried out laboratory experiments. ECT performed biological interpretation of the results. CC developed the mathematical model and performed mathematical interpretation of the results. MTN proposed the biological system to be modeled, designed *in vivo *and *in vitro *experiments and performed biological interpretation of the results. All authors read and approved the final manuscript.

## Supplementary Material

Additional file 1 **Supplemental Figure S1: *In vivo *ferritin's iron content in sucrose gradient fractions**. Caco-2 cells were grown in 7 cm^2 ^culture plates for 7 days in media containing 2, 4, 6, 10, 20 or 40 *μ*M of total iron. Cell homogenates were prepared as described and cleared by centrifugation (Arredondo *et al.*, 1997). Supernatants, corresponding to the cytosolic fraction of the cells, were loaded into a 1-25% sucrose gradient and centrifuged as described in Methods. Fractions were collected and ferritin determined by an ELISA assay as described (Arredondo *et al.*, 1997). Plotted is the ferritin content in the gradient fractions. Inserts: Ferritin migration into the gradient was transformed into Fe/ferritin mol/mol ratio utilizing data of the calibration curve. Note that the cells keep a fairly constant Fe/Fn ratio of 1,000 by increasing the total amount of ferritin: 3.2, 9.7, 52.4, 65.1, 78.3 and 83.3 ng of ferritin for 2, 4, 6, 10, 20 *μ*M iron in the culture. Shown 1 of 3 similar experiments. Click here for file
