# Supplementary figures and images for: Mathematical modeling of the dynamic storage of iron in ferritin
Source: BMC Syst Biol. 2010 Nov 3;4:147. doi: 10.1186/1752-0509-4-147 (PMC2992510; doi:10.1186/1752-0509-4-147)

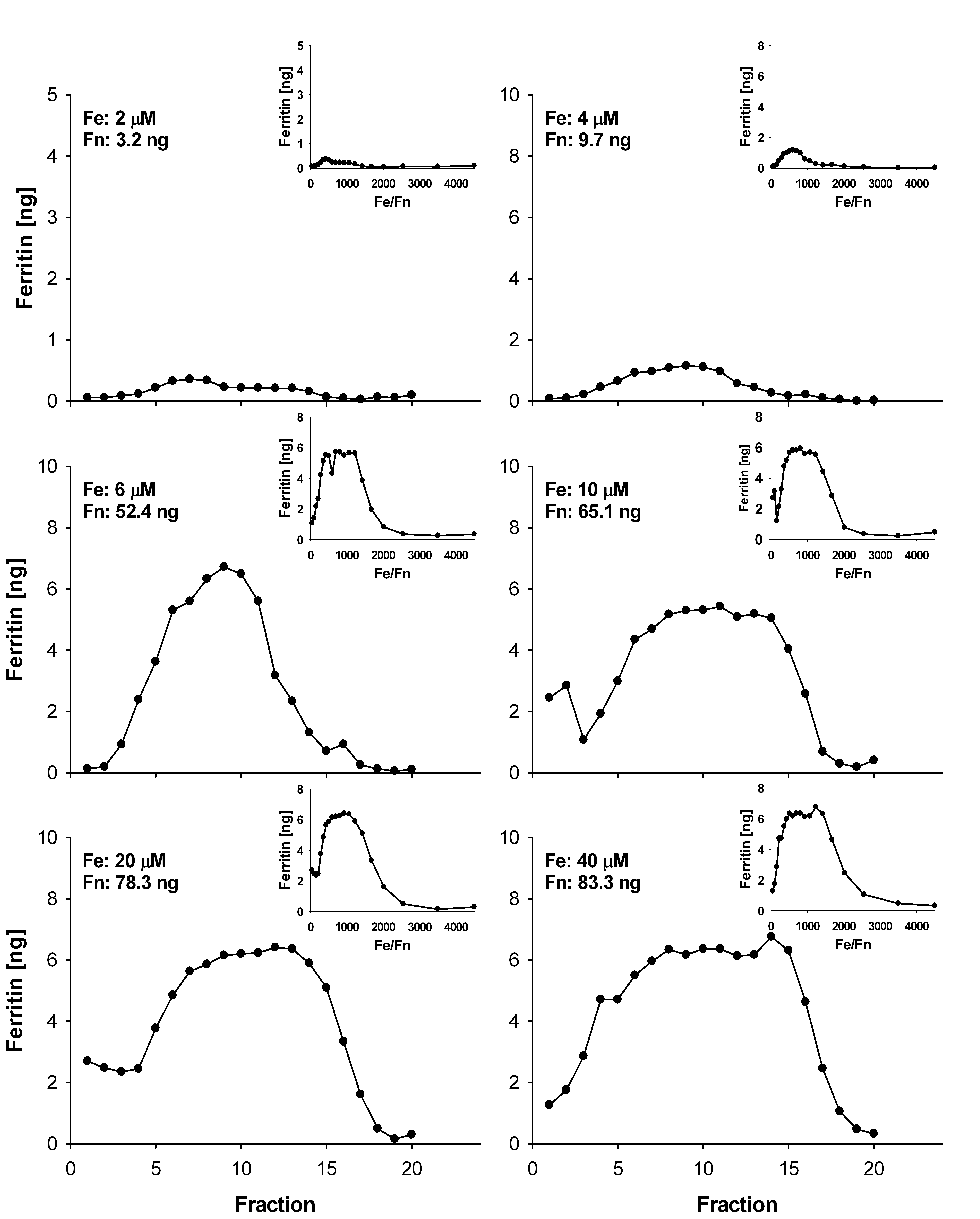

Supplement: Additional file 1 — Supplemental Figure S1: In vivo ferritin's iron content in sucrose gradient fractions. Caco-2 cells were grown in 7 cm2 culture plates for 7 days in media containing 2, 4, 6, 10, 20 or 40 μM of total iron. Cell homogenates were prepared as described and cleared by centrifugation (Arredondo et al., 1997). Supernatants, corresponding to the cytosolic fraction of the cells, were loaded into a 1-25% sucrose gradient and centrifuged as described in Methods. Fractions were collected and ferritin determined by an ELISA assay as described (Arredondo et al., 1997). Plotted is the ferritin content in the gradient fractions. Inserts: Ferritin migration into the gradient was transformed into Fe/ferritin mol/mol ratio utilizing data of the calibration curve. Note that the cells keep a fairly constant Fe/Fn ratio of 1,000 by increasing the total amount of ferritin: 3.2, 9.7, 52.4, 65.1, 78.3 and 83.3 ng of ferritin for 2, 4, 6, 10, 20 μM iron in the culture. Shown 1 of 3 similar experiments. [file 1752-0509-4-147-S1.TIFF]
